# Development of an LPS-based ELISA for diagnosis of *Yersinia enterocolitica* O:3 infections in Danish patients: a follow-up study

**DOI:** 10.1186/s12866-017-1035-1

**Published:** 2017-05-25

**Authors:** Tine Dalby, Eva Rasmussen, Peter Schiellerup, Karen Angeliki Krogfelt

**Affiliations:** Department of Bacteria, Parasites and Fungi, Statens Serum Institut, Artillerivej 5, DK-2300 Copenhagen S, Denmark

**Keywords:** Yersinia, Enzyme-linked immunosorbent assay, LPS, Antibodies, Human, Immunology

## Abstract

**Background:**

The bacterium *Yersinia enterocolitica* causes gastroenteritis in humans.

The study aimed to develop a diagnostic enzyme-linked immunosorbent assay (ELISA) for detection of *Yersinia enterocolitica* O:3 LPS antibodies in sera from Danish patients with suspected *Yersinia enterocolitica* O:3 gastrointestinal infection. As a part of this, antibody decay profiles after culture confirmed Yersinia enteritis were studied.

**Results:**

An ELISA using *Yersinia enterocolitica* O:3 LPS as the coating antigen was developed for measuring IgA, IgG and IgM specific antibodies. A longitudinal collection of 220 sera drawn between 20 and 1053 days after onset of symptoms from 85 adult Danish patients with verified Yersinia enteritis were examined. A control group of 100 sera from healthy Danish blood-donors were analysed in order to determine the cut-off for interpretation of results.

Serum samples from 62 out of 81 patients who delivered either the first or the second sample were found positive for specific antibodies against *Yersinia enterocolitica* O:3 LPS (77%). For samples collected within 60 days after onset of symptoms (*n* = 48) sensitivities of 58%, 42% and 79% for IgA, IgG and IgM antibodies were found. A sensitivity of 81% was found for these samples when using the definition of a positive result in either IgA, IgG or IgM as a combined positive. All samples received up to 36 days after onset of symptoms (*n* = 10) were found to be positive using this definition. For the period 61 to 90 days after onset of symptoms (*n* = 32), a combined sensitivity of 63% was found. The antibody levels as well as decay profiles for the three different immunoglobulin classes for the individual patients exhibited a large degree of variation.

**Conclusions:**

Using a definition of positive as a positive result for either IgA, IgG or IgM antibodies, a diagnostic sensitivity of 81% was achieved for samples received within 60 days after onset of symptoms. In particular, the levels of specific IgM antibodies were elevated. In comparison, the standard tube-agglutination assay achieved a sensitivity of 60% on the same samples. The sensitivity of the ELISA decreased the longer the duration of time since onset of symptoms. The ELISA was highly specific for Yersinia when testing sera from individuals with confirmed gastrointestinal infections by other bacteria. Moreover, the knowledge gained from this longitudinal study of antibody decay profiles can be used in future epidemiological studies of seroprevalence.

## Background

*Yersinia (Y.) enterocolitica* is an enteric pathogen, which causes an acute gastrointestinal disease in humans. *Y. enterocolitica* is widely distributed in nature in aquatic and animal reservoirs, with swine serving as a major reservoir for human pathogenic strains. Undercooked pork is frequently the cause of human Yersinia infections [1]. In the gastrointestinal tract, *Y. enterocolitica* can cause acute enteritis, enterocolitis, mesenteric lymphadenitis and terminal ileitis [2]. Furthermore, it is known that the bacterium plays an important role in the pathogenesis of reactive arthritis [3, 4].

Serological tests are valuable when the aetiological agent has not been isolated from a symptomatic patient and when the clinical symptoms indicate a previous infection. Furthermore, determination of specific antibodies can be important in the final diagnosis of sequelae after gastroenteritis such as arthritis and general joint pains [5].

A large community based study by Wheeler et al. investigated to what extent patients with gastroenteritis sought medical advice and had a stool culture performed [6]. It was shown, that the ratio between community incidence rate to general practice incidence rate varied from 1 to 2.1 for *Salmonella*, *Shigella*, and *Campylobacter* infections, whereas it reached 11.7 for *Yersinia* indicating a large extent of undiagnosed Yersinia infections. These results emphasize the need for valid serological tests for *Y. enterocolitica* antibodies.

Tube agglutination has long been used for serological diagnosis of *Y. enterocolitica* infections, but as agglutination is primarily dependent on IgM antibodies this method has been limited by low sensitivity especially if the response upon infection has consisted of mainly IgG or IgA [7]. Immuno-blotting and ELISA using Yersinia outer membrane proteins as antigens has also been used [8], yet it was found that these proteins cross-react with antibodies against *Borrelia*, *Salmonella*, *Brucella* and *Chlamydia* among others [9, 10]. Complement fixation has also been tried, but showed very low sensitivity [9]. There have been previous successful reports on measuring antibodies against Yersinia by ELISA using *Y. enterocolitica* O:3 lipopolysaccharide (LPS) as antigen for human diagnostics as well as veterinary use [11–15]. The use of species-specific LPS as antigen in an ELISA is a well-proven method for diagnosis of bacterial infections since LPS is highly immunogenic and moreover stable to work with in the laboratory [16–20]. There is sparse information on the course of development of antibodies in humans after a Yersinia infection, and previous longitudinal studies included very small numbers of patients [12, 13].

The aim of the study was to develop a diagnostic ELISA for detection of Yersinia enterocolitica O:3 LPS specific antibodies in humans. For this, sera from Danish patients with suspected Yersinia enterocolitica O:3 gastrointestinal infection were used. Furthermore, antibody decay profiles after culture confirmed Yersinia enteritis were assessed. Such knowledge on the decay of antibodies can be valuable for future studies of seroprevalence.

## Methods

### Serum samples

Sera from four groups (A-D) of subjects were included.

A: Ninety four Danish patients with a culture confirmed *Y. enterocolitica* O:3 infection were contacted. Participants were recruited from the Danish Registry of Bacterial Enteropathogens (DRBE) after a stool culture had confirmed bacterial gastroenteritis caused by *Y. enterocolitica.* All patients were asked to deliver a blood-sample 1 month after start of diarrhoea, as well as after three, six and 36 months. Nine patients did not return any samples. Seventy-seven patients delivered the first blood sample (82%), 39 (41%) delivered the second blood sample, 60 (64%) delivered the third sample, and 44 (47%) the fourth sample. The study was initiated in January 2002 and the last sample was taken in December 2004. There were 38 (40.4%) males and 56 (59.6%) females participating in the study and the median age was 43 years. There was a large variation in the intervals between samplings from the individual patients. A few of the patients (*n* = 15) did not report their start of symptoms, and this date was therefore set as the median difference from the date of the diagnostic fecal sample for culturing to the date of reported onset of symptoms for the remaining patients. This was minus 11 days (interval minus 2–160 days, mean minus 16 days).
B: Sixty three sera from patients with antibodies against *Y. enterocolitica* O:3 detected by tube agglutination.
C: Sera from 100 healthy Danish blood donors were analysed for determination of baseline levels of *Y.enterocolitica* O:3 LPS specific antibodies in the general population and thereby determination of a cut-off value for interpretation of the results.
D: Fifty eight sera from patients with serologically detected salmonellosis (18 sera from patients with confirmed antibodies against *Salmonella (S*) Enteritis LPS, 10 sera from patients with confirmed antibodies against *Salmonella (S)* Typhimurium LPS, 10 sera from patients with confirmed antibodies against *S.* Enteritidis flagellum, 10 sera from patients with confirmed antibodies against *S.* Typhimurium flagellum), and 10 sera from patients with serologically detected campylobacteriosis (confirmed antibodies against whole-cell *Campylobacter jejuni/coli* antigen). These 58 sera were included in order detect possible false positive reactions in the developed ELISA.

### Tube agglutination

*Y. enterocolitica* O:3 strain YeL was used for tube agglutination. Equal volumes of strain YeL in phosphate buffered saline (PBS) pH 7.38 were slowly mixed with 96% ethanol and left at 37 °C for 20 h. This stock solution was stable for approximately 4 weeks at 4 °C. Immediately before use, the stock solution was diluted in PBS to a final dilution of approximately 5 × 109 cells per ml.

0.3 ml of the final preparation of ethanol-killed cells were added to 0.2 ml of two fold dilutions of serum in PBS starting with a dilution of 1:25 and up to 1:3200. The results were read as the highest final dilution with granular agglutination after incubation for 20 h at 52 °C. A result of 1:400 and higher was evaluated as positive.

### ELISA procedure

An indirect ELISA was developed for determination of the content of specific antibodies against *Y. enterocolitica* O:3 LPS in human sera and inspired by method developed by Nielsen et al. for veterinary use [15]. Different combinations of microtiter plates, buffers and incubation times were evaluated and the most reproducible combination with low values for coefficient of variance (CV) was chosen. The ELISA was performed in microtiter wells [Nunc-Immuno™ plates PolySorp™ F96, ThermoFisher Scientific] and the analysis itself was automatized at a BioMek® 2000 robot [BeckmanCoulter Inc.]. Purified Yersinia 0:3 LPS [from a Danish porcine strain of Yersinia O:3 (BN5), The Danish Institute for Food and Veterinary Research prepared by acetone precipitation and phenol extraction according to previous reports [15, 21] was used as the coating antigen. The LPS was diluted to reach a concentration of 250 ng per ml in carbonate-buffer (at pH 9.60 containing 0.2% phenolred for monitoring of pH stability) and added at100μl to each well and incubated overnight at 4 °C. After a wash using PBS-Tween “wash-buffer” (phosphate buffered saline, PBS, at pH 7.40 containing 0.1% Tween®20 [Merck]), at 250 μl per well in 4 successive cycles, the wells were blocked by incubation with 250 μl of the wash-buffer per well for 30 min at room temperature. The wells were subsequently emptied, and 100 μl of serum diluted 400 times in PBS-Tween “dilution-buffer” (PBS at pH 7.40 containing 0.1% Tween®20 and 0.2% phenolred for monitoring of pH stability) was added per well in duplicates and incubated at room temperature for 30 min. After a wash of 4 cycles, 100 μl of dilutions made in dilution-buffer of horseradish-peroxidase conjugated rabbit anti-human either IgA, IgG or IgM [Dako, cat.no. P0214, P0215 and P0216] was added per well (anti-IgA diluted 500 times, anti-IgG diluted 2500 times and anti-IgM diluted 1000 times) and incubated at 30 min at room temperature. After a wash of 4 cycles, 100 μl of a tetramethylbenzidine solution (TMB) [Kem-En-Tec Diagnostics, cat.no. 4380A] was added per well and incubated for exactly15 minutes at room temperature. Finally, 100 μl of 1 M sulphuric acid was added per well, and the optical density at 450 nm minus the optical density at 630 nm was determined and used as the final result. Each serum was analysed for the presence of both IgA-, IgG- and IgM-anti LPS antibodies in three separate plates. Day-to-day variations were minimized by including a dilution-series of a standard as well as a positive and negative control at each analysis.

In order to avoid measurement errors, all tests were performed in duplicates and only results with a CV below 10% between the two samples were accepted.

## Results

### Determination of cut-off

In the 100 sera from healthy blood donors, optical densities (OD) obtained with the developed ELISA measuring IgA ranged from 0.00 to 0.31 (median 0.03), IgG ranged from 0.01 to 1.28 (median 0.07) and IgM ranged from 0.00 to 0.36 (median 0.04). The 95th percentile for IgA, IgG, and IgM was 0.31, 0.54 and 0.36 OD-values respectively. This 95th percentile of the blood donors was used as cut-off, and a specificity of at least 95% for the assay was thereby obtained. A positive result was defined as values at or above the cut-off.

### Sera positive by tube agglutination tested in ELISA

Sera (63) from patients with gastroenteritis and with a positive agglutination test against *Y. enterocolitica* were tested in the developed ELISA. The sensitivity was defined as the percentage of sera with detectable antibodies specific for *Y. enterocolitica* O:3 LPS, i.e. generating an OD-value at or above the cut-off. 90% (*n* = 58) had elevated levels of IgA class antibodies against *Y. enterocolitica* O:3 LPS, 60% (*n* = 38) had IgG class antibodies and 97% (*n* = 61) had IgM class antibodies. By combining all three antibody classes, the sensitivity of the ELISA compared to tube agglutination was 97%. Thus, tube agglutination assay is primarily successful in detecting antibodies of IgM class, less so for IgA end least for IgG.

### Sera from patients with confirmed *Yersinia enterocolitica* O:3 enteritis

**Fig. 1:**
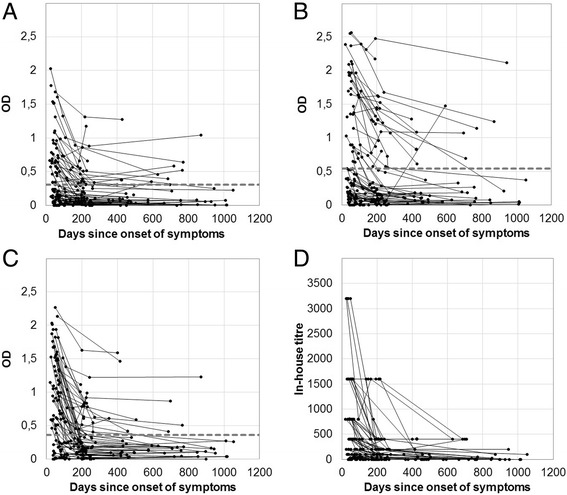
Serum antibody response to *Y. enterocolitica* O:3 infection in patients. **a** IgA; (**b**) IgG; (**c**) IgM, as well as agglutination titres (**d**). Each line represents a single patient. The dashed grey line indicates the cut-off values for each of the three classes of antibodies

From the 85 patients with a culture confirmed *Y. enterocolitica* O:3 infection, a total of 220 sera were received and subsequently analysed with the developed ELISA to determine their content of specific IgA, IgG and IgM antibodies. Serum antibody responses are presented in Fig. 1a-c.

The variation between patients is seen to be high, as some individuals had high values throughout the follow-up period, whereas some remained at low levels throughout the period. A few patients even had a second increase in levels of specific antibodies. Of the 81 patients, who delivered samples for either the first or the second sample (20 to 271 days after onset of symptoms), 62 individuals had elevated levels of specific antibodies of either IgA, IgG or IgM class, reaching a “combined sensitivity” of 77% (IgA: 47%, IgG: 41%, IgM:73%) while 19 (23%) did not show elevated levels. None other than these 62 individuals showed elevated levels of specific antibodies throughout the study period, i.e. 23 individuals (27%) did not show specific antibody-responses at the time when their samples were collected. No correlation was observed when comparing antibody-levels with age.

For samples collected up to 60 days after onset of symptoms (*n* = 48), a combined sensitivity at 81% was reached (IgA: 58%, IgG: 42%, IgM: 79%). For the period 61 to 90 days after onset of symptoms (*n* = 32), a combined sensitivity of 63% was found (IgA 28%, IgG 31% and IgM 63%). The combined sensitivity decreased with increasing time since onset of symptoms (Table 1). All samples received up to 36 days after onset of symptoms (*n* = 10) were found to be positive using this combined definition of positivity.

**Table 1:** Analysis of 85 sera from patients with a culture confirmed *Yersinia enterocolitica* O:3 infection

	Days (median)	No of samples	IgA	IgG	IgM	IgA, IgG, IgM combined	Agglutination
Sample 1	20–104 (53)	77	37 (48%)	30 (39%)	57 (74%)	59 (77%)	45 (58%)
Sample 2	66–271 (119)	39	12 (31%)	16 (41%)	14 (36%)	20 (51%)	13 (33%)
Sample 3	164–736 (209,5)	60	16 (27%)	19 (32%)	19 (32%)	29 (48%)	13 (22%)
Sample 4	353–1053 (610,5)	44	10 (23%)	10 (23%)	9 (20%)	18 (41%)	6 (14%)
Sample 1 and/or 2		81	38 (47%)	33 (41%)	59 (73%)	62 (77%)	47 (58%)
Up to 36 days	20–36 (29,5)	10	9 (90%)	4 (40%)	10 (100%)	10 (100%)	6 (60%)
Up to 60 days	20–60 (44)	48	28 (58%)	20 (42%)	38 (79%)	39 (81%)	29 (60%)
61–90 days	62–88 (71,5)	32	9 (28%)	10 (31%)	20 (63%)	20 (63%)	17 (53%)
3–6 months	93–182 (124,5)	36	11 (31%)	14 (39%)	12 (33%)	19 (53%)	11 (31%)
6–12 months	183–362 (213)	61	17 (28%)	21 (34%)	21 (34%)	31 (51%)	14 (23%)
1–2 years	377–719 (473)	26	7 (27%)	7 (27%)	6 (23%)	13 (50%)	6 (23%)
2–3 years	736–1053 (896)	17	3 (18%)	3 (18%)	2 (12%)	4 (24%)	0 (0%)

Number and percentages of sera producing OD-values above the cut-off when testing in an ELISA using *Y. enterocolitica* O:3 LPS as antigen or with tube agglutination. Patients delivered up to 4 samples following culture confirmation of *Y. enterocolitica* infection. No patient donated a sample for every one of the four presented time ranges

Age and sex were found to have no influence on the magnitude of the antibody-response.

### Tube agglutination

The tube agglutination test was performed for all the collected sera and compared to ELISA (Table 1 and Fig 1d). Of the 81 patients, who delivered samples for either the first or the second sample (20 to 271 days after onset of symptoms), 47 individuals (58%) had agglutinating antibodies while 34 (42%) had not. When analysing the samples received up to 36 days after onset of symptoms, that were all found positive in the ELISA test (*n* = 10), 6 were found to be positive by tube agglutination (60%).

For the period of up to 60 days after onset of symptoms (*n* = 48), 60% of the samples were found to be positive for agglutinating *Y. enterocolitica* antibodies. For the period 61 to 90 days after onset of symptoms (*n* = 32) the sensitivity dropped to 53%. Thus, the ELISA showed a higher sensitivity than tube agglutination.

### Cross-reactions

Sera from patients with serologically confirmed infection due to other bacteria causing gastrointestinal infections were assayed for reactions in the developed ELISA using the cut-offs for IgA, IgM and IgG, as described above.

Serum samples from patients with reacting antibodies to *S.* Enteritidis, *S.* Typhimurium or *Campylobacter jejuni/coli* were tested for cross-reactions in the developed ELISA. Among the serum samples from patients with antibodies specific for *Salmonella* Enteritidis flagellum, *S.* Typhimurium flagellum, *S.* Typhimurium LPS or *Campylobacter jejuni/coli* - none gave reactions in the ELISA. Among the serum samples from patients with antibodies to *Salmonella* Enteritidis LPS (*n* = 18), three samples (16.7%) were positive; two just for IgG and one for both IgA, IgG and IgM.

## Discussion

In a patient population suffering from gastroenteritis due to *Y. enterocolitica* O:3, we found a high prevalence of specific antibodies against *Y. enterocolitica* O:3 LPS by the newly developed ELISA assay. The antibody responses for 85 patients were followed for more than a year after infection in order to evaluate the usefulness of the analysis in relation to time since onset of symptoms. When serology is used as a diagnostic tool, interpretation of results can be difficult unless such decay profiles are established. There are two previous reports on such decay profiles, but these were carried out with just 9 or 15 patients [12, 13].

At up to 36 days after onset of enteric symptoms, 10 of the 10 received samples (100%) were found positive for the presence of specific antibodies of either IgG, IgM or IgA class. The number is too low to make a solid conclusion, but it is promising with respect to early diagnosis. In comparison, the sensitivity obtained by standard tube agglutination for the same 10 samples was 60% (*n* = 6). The sensitivity of the ELISA analysis decreased with time since onset of symptoms, but remained higher than the sensitivity for tube agglutination.

It is intriguing that the immune response and the different antibody classes have such great variation between the patients. Previous infections with *Y. enterocolitica* might play an important role in this variation. Age and sex were found to have no influence on the magnitude of the antibody-response. The study is complicated by the fact that the patients donated samples at widely varying time-points. Moreover, for a few patients, a later increase in the levels of specific antibodies during the course of the study might imply an additional *Yersinia* infection during the study period (Fig 1). However, when looking only at the levels found for samples donated in the beginning of the study, it is clear that the levels of antibodies decrease quite rapidly for all three classes of antibodies. Thus, it is important for the interpretation of results to compare with the time passed since onset of symptoms. Moreover, this will pose some limits to the use of the assay for post-infection diagnostics or diagnostics of reactive arthritis thought to be due to a previous *Yersinia enterocolitica* O:3 infection.

The specificity of the ELISA was high when tested against samples from patients infected with other gastrointestinal pathogens. Three out of 18 individuals with confirmed antibodies against *S. enterocolitica* LPS did however have a positive result by the developed ELISA. Due to the inconsistency of this, these three individuals could likely have had a recent infection by *Yersinia enterocolitica*. No details of the history of previous gastrointestinal infections for the participating individuals are known.

The *Y. enterocolitica* O:3 LPS based ELISA is superior to the standard tube agglutination assay, which primarily detects IgM antibodies in the acute phase. Previous reports on the use of LPS for ELISA were performed on selected agglutination-positive sera in contrast to this study where sera from culture confirmed cases were used. Our study therefore allows for a comparison of sensitivity for agglutination and ELISA that could not be determined in the old studies [11, 12].

With the knowledge of the decay of antibodies after an *Y. enterocolitica* infection, the ELISA can moreover be used for sero-epidemiological studies, as has previously been performed for Campylobacter and *Salmonella* infections [22–24].

## Conclusion

An in-house ELISA for detection of specific antibodies against *Yersinia enterocolitica* O:3 LPS in human sera was developed and found to be superior when compared to the standard tube-agglutination assay. Diagnostic cut-offs for IgA, IgG and IgM were determined based on the 95th percentile of levels in sera from healthy Danish blood-donors. For samples received within 60 days after onset of symptoms, a diagnostic sensitivity of 81% was achieved. The sensitivity decreased the longer the duration of time since onset of symptoms. A high specificity was achieved when analysing sera from individuals with confirmed gastrointestinal infections by either *Salmonella* or *Campylobacter*. The developed method can be used for diagnostic purposes as well as for sero-epidemiological studies.

## Abbreviations

CV: Coefficient of variance
DRBE: Danish registry of bacterial enteropathogens
ELISA: Enzyme-linked immunosorbent assay
IgA: Immunoglobulin A
IgG: Immunoglobulin G
IgM: Immunoglobulin M
LPS: Lipopolysaccharide
O:3: O chain polysaccharide antigen type 3
OD: Optical density
PBS: Phosphate buffered saline
SSI: Statens Serum Institut
TMB: 3,3′,5,5′-Tetramethylbenzidine
